# Extrauterine Fœtation

**Published:** 1901-08-10

**Authors:** 


### Hospital Clinics and Medical Progress.

### EXTRAUTERINE FŒTATION.

In an address delivered before the Watford Medical Society, Mr. John D. Malcolm, surgeon to the Samaritan Free Hospital, after describing the course and complications of extrauterine fœtation, dealt with the subject of diagnosis. The evidence pointing in the direction of an extrauterine fœtation may, he said, be placed in two divisions; first the history, second the physical conditions found. As to history, the patient must of course be in the child-bearing period. It has been noted that an extrauterine gestation is not infrequently preceded by a long interval of sterility in a woman who has already borne children, but this is not a very important matter, for this accident may apparently occur at any time and in apparently healthy women. The symptoms which accompany an extrauterine gestation going on to the full term may be exactly those of a normal pregnancy, but the very great enlargement of the blood supply which is an invariable accompaniment of the condition must favour the occurrence of a discharge of blood from the uterus, and thus, even when the fœtus is developing without serious trouble, the periods usually come on, although perhaps with a little delay. There may be two or three months missed, but more commonly the patient goes a week or two beyond her time and then has a more or less constant loss, or a frequently recurring uterine flux of a few days' duration may be noted. A deciduous membrane forms in the uterus when an extrauterine conception occurs, and is thrown off at some period of the gestation, either as a complete cast of the uterus or in shreds of varying size. It often separates about the end of the third month, and then the patient may suppose that she has miscarried and may assert as a fact that she has done so. This discharge of the decidua may coincide with the rupture of the tube and the death of the embryo. Thus, so far as history is concerned, the points which usually attract attention are a slight delay in menstruation followed by irregularity, in a patient who has been previously regular. If, in addition to this, after two or three months a deciduous membrane is passed (the woman never having passed such a thing before), an extrauterine pregnancy may be diagnosed almost with certainty from the history alone, and if milk appears in the breasts the presumption is rendered stronger still. It must be remembered that there may be no pain and no other trouble of any kind.

On examining a case of extrauterine fœtation an abnormal mass will be found in the pelvis, or in both pelvis and abdomen, with the uterus separate from it, or, in case the fœtus has developed in the portion of the Fallopian tube which lies within the walls of the uterus, this organ might not be felt separate but would probably form, together with the gestation, an irregular mass which would be unlike the ordinary pregnant uterus. It might, however, be very difficult to differentiate such a condition from a normal pregnancy with accidental hæmorrhages, but, excluding this condition and assuming the child to be alive, the mass would correspond to the growth of the fœtus, dating from about the time of the first irregularity of the menstruation.

In the later months the fœtus would be distinguishable by palpation, its movements might be more obvious than in the normal condition, and they are often described as being more painful. Beyond these there may be no symptoms other than might occur in normal pregnancy. In some cases, however, there may be severe pain due to pressure and traction, while in some it may be almost impossible to define the fœtus by palpation on account of its relations to the intestines. At full term a spurious labour may take place and the patient may perhaps not be seen by a medical man until after the child is dead.

In the more common form in which the placenta becomes detached, the practitioner is usually called in in connection with the symptoms arising from the hæmorrhage. The sudden hæmorrhage into the peritoneal cavity or connective tissue gives rise to acute pain with vomiting, quick pulse, faintness, and pallor, the last three being in proportion to the amount of blood lost. If an examination be made immediately after the first hæmorhage a boggy swelling in the pelvis may be found, more or less free from the uterus if the blood has escaped into the peritoneal cavity; but if it is in the connective tissue it will push over and fix the uterus, pressing close up to its walls. Blood free in the cavity, however, also becomes a definite firm mass when an adventitious membrane forms around it. If the patient be kept quiet and no fresh hæmorrhage occur there is usually some elevation of temperature and pulse for a few days; but the tendency is for the blood to be absorbed whether it be intra- or extra-peritoneal. But fresh hæmorrhages may take place, each of which may cause an alteration in the size and shape of the tumour, which may become very large. With such a history and with such physical condition signs there should be no hesitation in diagnosing an extrauterine gestation.

But sudden and profuse internal hæmorrhage may occur very early in the case, so early that there may be very little history to be obtained, or the patient may, in consequence of the hæmorrhage, be in such a condition as not to be able to give any information. She may have appeared to be in good health until she was seized with pain, and on examination there may be but little to be perceived. The blood being fluid cannot be felt, and the ruptured tube being depleted, the remains of conception may be soft and difficult to define. Hence, in these very dangerous cases one must go chiefly by the symptoms, which are:—Acute pain in the abdomen, faintness perhaps to the point of insensibility, profuse perspiration, breathlessness, increasing restlessness, a rapidly rising pulse rate, and increasing pallor. It may be very difficult to tell whether the condition is due to hæmorrhage or to the shock of such an accident as the rupture or strangulation of the bowel, but there is a steady progression of the adverse symptoms in the case of hæmorrhage which is very characteristic. There may be other symptoms which may help in the diagnosis, such as the discharge of a deciduous membrane or the history of a little irregularity of menstruation, or there may be a little swelling at one side of the uterus, but the medical man may find little or nothing to guide him to a diagnosis except the symptoms of hæmorrhage.

If a profuse hæmorrhage is diagnosed no avoidable delay in resorting to surgical interference is justifiable. Nothing else will save the patient, and she may almost certainly be saved by prompt removal of the affected tube. The worse she is the more urgent is the necessity for arresting the hæmorrhage. Saline transfusion should, of course, be employed at the same time if necessary. When the hæmorrhage is not so severe complete rest in bed may allow absorption of the blood, but where there are evidences of repeated hæmorrhages it may be better to operate at once, and whenever the child is believed to be alive the proper treatment is to remove it without delay. This latter dictum may not meet with universal acceptance, but Mr. Malcolm has no hesitation in saying that to allow a living fœtus to go on developing outside the uterus will in a very great majority of cases, if not in every case, expose the patient to greater risks than those of an operation.

